# Tubercular Affection of the Larynx

**Published:** 1890-11-22

**Authors:** 


### The Practitioner's Mirror and Retrospect.

[*The Editor will be glad to receive offers of co-operation and contributions from members of the profession. There is no doubt that much valuable material full of interest and instruction is at present lost to science. This is largely so in the case of* (1) *the younger and less-known members of the hospital staff* (2) *those connected with cottage hospitals, and* (3) *the great body of medical practitioners not attached to any institution. The co-operation of all is cordially invited to enable the Editor to make* The Hospital *of the utmost possible use and value to the profession. Specialists willing to review books on their special subjects are invited to communicate this fact at once. All letters should be addressed to* The Editor, The Lodge, Porchester Square, London, W.]

### THE POST-GRADUATE COURSE AT THE BROMPTON CONSUMPTION HOSPITAL.

### TUBERCULAR AFFECTION OF THE LARYNX.

Dr. Percy Kidd said*:—In commencing the subject of tubercular affections of the larynx it will be well to give a brief description of the anatomical parts of this organ. The general anatomy of the cartilages, muscles, nerves, and vascular supply is fully recognized, but there are some points with regard to the mucous membrane that are worthy of notice. The epithelium covering the vocal cords and the epiglottis is laminated pavement epethelium, that of the other parts of the larynx being ciliated columnar. So also in most parts of the larynx the submucous tissue is loose, but that of the vocal cords is more compact and contains many dense bundles of elastic tissue. This form of tissue occurs in other regions of the organ, but only in isolated fibres. The tubercular lesions of this region are of two kinds, (1) infiltrations or deposits, and (2) ulcerations. The first form may occur either as isolated nodules or as a diffuse infiltration, and the nodules may be miliary or large deposits, in some cases forming tubercular tumours. The deposit consists in either case of a cellular growth, not differing in any degree from the similar process occurring in other mucous membranes of the body. Tubercle, in fact, is a cellular formation with a certain structure, and liable to undergo certain changes such as caseation and fibrosis. In the second variety the characters of the ulcer are as follows: The bases are irregular and necrotic, the edges are irregular, thick, and often sinuous; the parts about are pale, the process is slow; the ulcers may be solitary, but they usually appear on more than one spot; the sinuous type of ulceration ends with a coalescence of many tubercular foci. The secondary results of this disease are œdema, degeneration of the muscles, and necrosis of the cartilages. Localisation: The most usual seat of the disease is the vocal cords, and especially the posterior extremity of these and the posterior wall of the larynx; that is to say, the interarytenoid folds. Here ulceration is usually seen, but this is probably preceded by infiltration, and this condition is most likely due to the arrangement of the structures of the mucous membrane in this part. The tubercular process extends in the region of least resistance. In an early stage the deposit is seen lying immediately beneath the mucous membrane, and from here it extends towards the free surface, and not into the deeper parts, on account of the firm resisting layer of elastic tissue in the deeper structures, and hence ulceration of the cords is commoner than infiltration. In some cases there is a circumscribed and in others a diffuse infiltration of the cords, in which case they appear of a pale red colour and often very irregular; sometimes they become two nodular bands, all resemblance to the original cords being lost. At first the deposit or ulcer is most commonly unilateral or more advanced on one side than on the other. On the posterior wall of the larynx, infiltration is often recognised, and this is followed by ulceration, and, owing to the looseness of the tissues here, these ulcers are cup-shaped, the edges are jagged and raised; the ulcers on the cords on the other hand are usually much thickened with their characteristic shallow oval or concave appearance. The ulcers on the cords commonly occur on the upper surface towards the posterior part at the processus vocalis, the long axis of the ulcer corresponding to the length of the cord. In other cases the ulcers may split the cord into two segments, and produce the appearance of two vocal cords one above the other, and occasionally in the same way many may be formed. In an advanced stage complete effacement of the cords is met with, or the vocal cord and the interventricular band may be fused. The aryteno-epiglottidean folds are rarely affected except from extension from the neighbouring parts. On the epiglottis the lesions consist of shallow or deep ulcers on the laryngeal surface, or in some cases there are ulcers with much thickening; sometimes there is considerable swelling prior to ulceration in this region. So also there may be complete destruction of the epiglottis, which is usually described as pathognomonic of syphilis. The false cords are very much less liable to become affected than the true ones. The following statistics were then given as to the localisation of the disease from the post-mortem reports in a hundred cases of tubercular laryngitis. Of these hundred, in 23 the lesions were so widespread that they had to be excluded for the purpose of localisation. Thus, from the 77 cases left, in 53 the vocal cords were affected (*i.e.*, 68 per cent.); in 47 the arytenoids and posterior wall of the larynx (*i.e.*, 61 per cent.); in 21 the epiglottis (*i.e.*, 27 per cent.); and in four the ventricular bands (*i.e.*, 5 per cent.). Occasionally there were deposits in the sub-glottidean regions, but only in advanced cases. As the result of the disease on the cartilages perichrondritis and necrosis sometimes occur, especially the latter, as the result either of deep-seated infiltration or from deep ulceration, and portions of the necrosed cartilages may be expectorated. The changes about the arytenoids are of importance because of the alterations these produce in the cords or in modifying their movements, and as the result one of the cords may become fixed, either in adduction or in abduction, and in which of these two positions this occurs makes a very considerable difference to the patient. Degenerative changes occurring in the muscles as the result of tubercular affections are not common. The œdema which often results is always secondary, and is usually combined with infiltration. In speaking of the disease, laryngeal tuberculosis is now used instead of the older term, laryngeal phthisis, it now being established beyond all doubt that they are the same affections. Various causes for the ulcers have been described—as from deposits in glands, follicles, &c.—but probably the disease is from the beginning a tubercular specific one. No age is free from the affection, but it is more common in young middle life, also more common in the male than in the female. With regard to the question of the occurrence of a primary laryngeal tuberculosis, a short time ago, among laryngologists it was considered a very common occurence, and among general physicians equally rare. More recently a few cases have been recorded of the disease first appearing in the larynx, but it is commoner by far as a secondary affection to a primary pulmonary tuberculosis, and this is seen not only from post-mortem examinations, but also clinically. In these cases of laryngeal mischief it is often exceedingly difficult, and in fact, in some cases impossible to be able to say definitely whether there is

* Our best thanks are due to Dr. Percy Kidd for permitting us to give a summary of his lecture, especially as it was not prepared for publication.

pulmonary disease or not, as frequently there are no signs to be obtained, many of the usual auscultatory methods not being applicable, on account of the absence or changes in the voice of these patients. On the question of the methods of infection we have two views, one—that it is a localised infection from the sputum of the diseased lungs, and the other, that the germs come through the blood vessels, and produce a disseminated tuberculosis. One observer has suggested that the sputum has a corrosive action, and so gives rise to ulceration. Occasionally laryngeal tuberculosis occurs in cases of disseminated tuberculosis, but usually it appears to be the result of the contamination from the sputum, for the following reasons: The nature of the sputum and the co-existence of pulmonary and laryngeal tuberculosis without disease in the other organs of the body; so also the localisation of the disease in the larynx itself, that is, on the posterior part of the cords and on the posterior wall of the larynx, all of those parts whose epithelial covering is non-ciliated. Where the cords are inserted there is a possibility of some friction between them during the movements of phonation, and this would produce ulceration. Another suggestion is that the sputum becomes arrested in the ventricles, and from here overflows on to the cords, and so starts the process; but the ventricles being lined with ciliated epithelium, it would not appear that sputum would have much opportunity of remaining in this position long, but it must be perpetually moving on. The chief objection that has been raised to the inoculation theory is, that in some cases from the first there appears to be a deep-seated infiltration, and not the condition one might expect from a superficial infection. But even in these cases it is not necessary to assume the presence of a primary sore, as we know in other parts infection may occur without leaving any trace of the spot of its primary entrance. The symptoms are the following: (1) *Paræsthesia;* the same as of any laryngeal disease, such as alteration in sensation, tickling in the throat, the sensation of a foreign body, burning. (2) *Phonation;* hoarseness or loss of voice (aphonia), and this may be due to two classes of causes (*a*) mechanical, (*b*) nervous influences, paresis. (3) *Deglutition;* great pain in swallowing, especially if there are changes about the epiglottis or about the posterior wall of the larynx. (4) *Respiratory;* a cough which may be laryngeal in origin. (5) *Expectoration* may also come from the secretions from the laryngeal ulceration. (6) *Dyspnœa;* this occasionally is not pulmonary but laryngeal, especially if there is much obstruction of the glottis, so, also, if one cord is prevented from closing from any cause, either from a mechanical or nervous lesion; in these cases the opposite cord tries, but fails to meet its fellow with each movement of phonation. The patient thus has to use more breath than normal, and this has been called the "phonative waste of breath." (7) *Circulatory* hæmorrhage, but this, to any extent, is very rare in this affection.

The following are the appearances of the morbid parts: First, the vocal cords: in an early stage a number of distended vessels are seen, and later a loss of substance on the upper surface or on the edge of the cord. The cords then become red throughout, and lose their glistening appearances; in other cases the whole cord becomes greatly thickened, has a fleshy appearance, and from this there may follow complete destruction of the cords. In these cases the patients are still able to phonate, although there is complete absence of the original vocal cords, and this is done through the ventricular bands taking on the function of phonation. When the posterior wall of the larynx is affected, the commonest appearance is swelling with the appearance of a sort of pad, which fills up the inter-arytenoid fold and projects between the cords. This consists not only of an infiltration, but also frequently of the thickened edge of an ulcer, the floor of the ulcer not being seen on account of its vertical position, or at other times a superficial ulcer may be seen in this region.

The epiglottis may have distinct ulcers on its surface, or a condition of great thickening of the whole epiglottis, which structure may be in these cases folded backwards, and so hide the rest of the larynx from view. The ventricular bands may be thickened and reddened, in some cases completely obscuring the cords. The course of the disease varies much. Generally it is gradual and progressive, arrest is sometimes obtained in even what appear to be unfavourable cases. And there is no doubt that even cure is occasionally but very rarely obtained. The duration of life greatly depends upon the general condition of the patient; if there be much pain in deglutition there quickly ensues a rapid failure in nutrition.

